# Association of finerenone with prognosis and safety in diabetic kidney disease patients: an undated meta-analysis based on four RCTs

**DOI:** 10.3389/fmed.2025.1594202

**Published:** 2025-07-03

**Authors:** Zixuan Zhang, Fan Zhang, Yan Bai, Jiao Li, Yifei Zhong

**Affiliations:** Department of Nephrology, Longhua Hospital Shanghai University of Traditional Chinese Medicine, Shanghai, China

**Keywords:** diabetic kidney disease, finerenone, mortality, systematic review, meta-analysis

## Abstract

**Background:**

Although current guidelines have recommended finerenone as a first-line agent for patients with diabetic kidney disease (DKD), it is unclear what effect finerenone has on all-cause and cardiovascular mortality. This study aimed to assess the impact of finerenone on the prognosis and safety of patients with DKD.

**Methods:**

A systematic search was performed in PubMed, Embase, Scopus, and Web of Science. We included randomized controlled trials involving patients diagnosed with DKD that had finerenone versus placebo. The number of deaths, including any cause and cardiovascular causes, hyperkalemia, and adverse events, were collected for the finerenone and placebo groups. Data were summarized as risk ratio (RR) with 95% confidence interval (95% CI).

**Results:**

Four trials (13,943 participants) were included in the meta-analysis. Results of the restricted maximum likelihood-adjusted random-effects model showed that finerenone was associated with a reduced risk of all-cause (RR: 0.894; 95% CI 0.802–0.998) and cardiovascular mortalities (RR: 0.824; 95% CI 0.685–0.990) in DKD patients. Finerenone predisposed to hyperkalemia compared with placebo (RR: 2.280; 95% CI 1.937–2.682).

**Conclusion:**

This meta-analysis provides key information on the prognosis and safety of finerenone in DKD patients. These results help to supplement the clinical evidence for finerenone.

**Systematic review registration:**

https://www.crd.york.ac.uk/PROSPERO/, CRD42023463227.

## Introduction

Diabetes mellitus is the leading cause of chronic kidney disease (CKD), and about 30%–40% of diabetic patients develop CKD (1). The number of new cases of CKD due to type 2 diabetes mellitus has been reported to have increased globally from approximately 1.4 million cases in 1990 to 2.4 million cases in 2017, an increase of 74% (1). Globally, 15%–50% of end-stage renal disease is caused by diabetic nephropathy (2, 3). It is characterized by glomerular filtration barrier dysfunction and decreased renal function, which can be directly reflected by persistent elevation of urinary albumin and progressive decrease in estimated glomerular filtration rate (eGFR), respectively (4).

Diabetic kidney disease is associated with excess all-cause and cardiovascular mortality in patients with diabetes (5). Surveys from the United States have shown that the 10-year cumulative standardized mortality rate rises from 7.7% in patients without diabetes/nephropathy to 11.5% in patients with type 2 diabetes mellitus but no nephropathy and then to 31.1% in patients with type 2 diabetes mellitus and nephropathy (6). In addition, the risk of cardiovascular mortality is statistically at least three times higher in DKD patients than in diabetes mellitus patients without CKD (7).

Finerenone, a novel non-steroidal mineralocorticoid receptor antagonist with higher receptor selectivity than spironolactone and better receptor affinity than eplerenone *in vitro*, provides cardiorenal and renal dual-protective effects in DKD patients (8). In randomized controlled studies, finerenone reduced proteinuria in patients with CKD and heart failure with a lower incidence of hyperkalemia than spironolactone (9). Thus, finerenone may meet the medical need to safely manage proteinuria without affecting blood potassium in patients with DKD. However, in another double-blind trial, it was observed that the incidence of interruption of the trial protocol due to hyperkalemia was higher in the finerenone group than in the placebo group (10).

To date, four meta-analyses have focused on the therapeutic effects of finerenone in DKD patients (11–14). However, these studies either used non-placebo controls (11), did not consider “zero-event” studies (13), or lacked calculations of optimal information (12). Although current guidelines have recommended finerenone as a first-line agent for patients with diabetes mellitus combined with CKD (15), its effects on all-cause and cardiovascular mortality remain under discussion (16). To assess the prognosis and safety of finerenone more scientifically for treating DKD, this study used meta-analysis to analyze the situation of DKD patients after using finerenone to provide evidence-based references for the drug’s clinical use.

## Method

### Protocol and guidance

This systematic review and meta-analysis were conducted following the 2020 Preferred Reporting Items for Systematic Reviews and Meta-Analyses (PRISMA) guidelines (Supplementary Table S1) (17). The protocol has been registered in the International Prospective Register of Systematic Reviews (PROSPERO) with CRD42023463227. The current study methodology is like the previously described protocol with a few modifications (Supplementary Table S2).

### Data sources and search strategies

We systematically searched four electronic databases (Embase, PubMed, Web of Science, and Scopus) using a related keywords, including finerenone, diabetic kidney disease, and also searched ClinicalTrials.gov^1^ and World Health Organization (WHO) International Clinical Trials Registry Platform (ICTRP)^2^ for ongoing studies. The timeline was from the inception to June 01, 2024. The search strategies for each database are shown in Supplementary Table S3.

In addition, we also manually searched for records in the reference lists of previous systematic reviews. Literature downloaded from the databases was imported into EndNote 20 software for management.

### Eligibility criteria

Eligibility criteria were based on PICOS (Population, Intervention, Comparator, Outcome, Study design) elements developed by potential randomized control trial (RCT) should include (1) adults with a diagnosis of diabetic kidney disease (both secondary and primary included), whether pre-dialysis, dialysis dependent, or kidney transplant recipients, (2) participants in the exposure group receiving finerenone treatment, (3) participants in the control group received a placebo treatment, (4) primary outcomes of interest is all-cause mortality. Secondary outcomes were cardiovascular mortality, which was defined as death from cardiovascular causes, hyperkalemia related to a trial regimen, and any adverse events.

### Selection process

Two authors (ZXZ and YB) independently conducted the selection. The titles and abstracts were screened for relevance based on eligibility criteria, and potential full-text articles were reviewed. Disagreements were adjudicated by a third author (FZ).

### Data extraction

Two independent reviewers (FZ and ZXZ) extracted data using an established form, including first author, publication year, dose of finerenone, age, gender, duration of treatment, serum potassium and eGFR at baseline. If a randomized controlled trial (RCT) has more than two treatment groups, we pool the data from the different treatment groups. Any discrepancies were resolved through discussion.

### Risk of bias assessment

Two independent authors (ZXZ and YB) assessed the risk of bias for each included RCT according to the Cochrane Collaboration’s risk of bias tool 2 (RoB-2) as having a low risk of bias, some concerns or a high risk of bias (18). The Excel macro tool provided on the RoB-2 official website^3^ was used to generate the risk of bias summary table. Any discrepancies were resolved through a third author (FZ).

### Data analysis

We performed statistical analyses using the meta (19) package in R (version 4.2.0; R Project for Statistical Computing). Dichotomous variables were extracted as absolute numbers and percentages. Results were summarized using the Mantel-Haenszel method and presented as risk ratios (RR) and 95% CIs. We added a 0.5 correction factor to the calculation for studies reporting zero events. Considering sample and geographic differences, all analyses used random effects models with restricted maximum likelihood (REML) variance estimates. For the primary outcome (all-cause mortality), we calculated the number need to treat (20).

Furthermore, 95% prediction intervals were calculated to forecast the actual effects range (21). Publication bias and statistical heterogeneity between studies were assessed by visual inspection of forest plots and I^2^ statistics for heterogeneity (22, 23). The total effect *z*-test determined the significance level of the treatment effect. The significance level for the treatment effect was defined as two-sided *P* < 0.05. Due to the limited number of included studies (< 10 per variable), meta-regression and funnel plot asymmetry tests were not performed. We assessed the robustness of the results using a “leave-one-out” approach and excluding “zero-event” studies, respectively.

### Trial sequential analysis

We performed a trial sequence analysis to explore whether the cumulative data had sufficient power to assess outcomes, setting an overall 5% risk of type I error and 80% power (24, 25). Our initial anticipated intervention effect was a 10% relative risk reduction. In any adverse event analysis, we used a reduced threshold (5%). The analysis was performed using trial sequence analysis software (version 0.9.5.9 Beta).

### Grading of evidence assessment

Two independent reviewers (YB and ZXZ) assessed the cumulative evidence for each outcome using the Grading of Recommendations, Assessment, Development, and Evaluation (GRADE) framework. The certainty of evidence was rated as high, medium, low, and very low by the GRADE tool (26).

## Results

**FIGURE 1:**
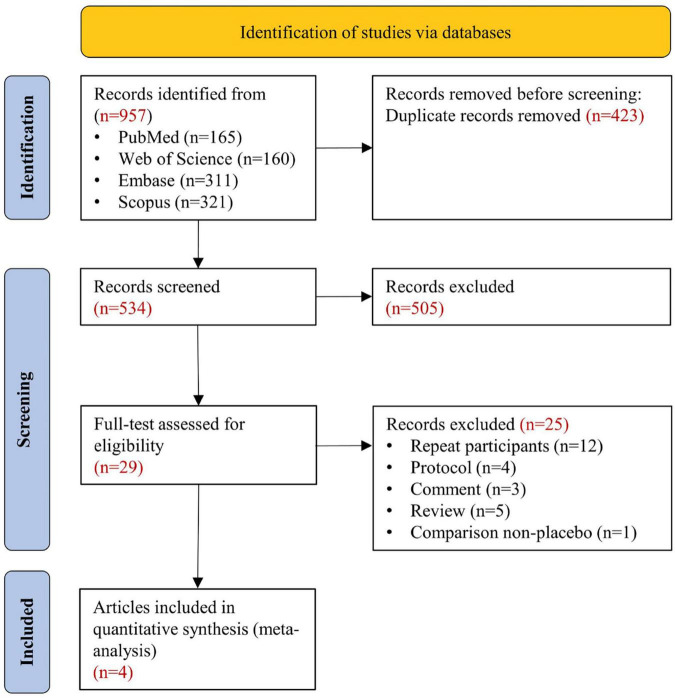
Search strategy and final included and excluded studies.

Figure 1 shows the systematic search and study screening process. After excluding 423 duplicate records and an additional 505 records that did not meet the inclusion criteria, we read the full text of 29 records; of these, four RCTs were deemed to meet the inclusion criteria (10, 27–29). Table 1 describes the details of included trials. These studies had a total of 13943 participants, 1166 deaths from all causes, and 686 deaths from cardiovascular disease. Supplementary Figure S1 shows a risk of bias on four included RCTs. Table 2 summarizes the details of four large ongoing clinical trials.

**TABLE 1:** Characteristic of included studies.

References	Publication year	Country	Groups	Participants (Male/Female)	Age, years	eGFR at baseline, mL/min/ 1.73 m^2^	Urinary albumin- to-creatinine ratio at baseline, mg/g	Serum potassium at baseline, mmol/L	Duration
Bakris GL et al. (10)	2020	International	Placebo	2841 (2030/711)	65.7 ± 9.2	44.3 ± 12.6	867 (453–1645) ^1^	4.38 ± 0.46	2.6 years
			Finerenone 10 or 20 mg	2833 (1953/880)	65.7 ± 9.2	44.4 ± 12.5	833 (441–1628) ^1^	4.37 ± 0.46	90 days
Bakris GL et al. (29)	2015	International	Placebo	94 (69/25)	63.26 ± 8.68	72.2 ± 20.4	182.9 (15.0–3056) ^2^	4.30 ± 0.48	
			Finerenone 1.25 mg	96 (78/18)	64.91 ± 9.57	66.1 ± 21.9	216.8 (14.1–2707) ^2^	4.32 ± 0.43	
			Finerenone 2.5 mg	92 (78/14)	64.86 ± 9.09	67.4 ± 20.2	158.9 (21.2–4020) ^2^	4.30 ± 0.43	
			Finerenone 5 mg	100 (71/29)	63.31 ± 8.79	67.1 ± 22.2	174.8 (27.9–2649) ^2^	4.31 ± 0.33	
			Finerenone 7.5 mg	97 (79/18)	63.73 ± 10.04	67.5 ± 21.9	166.4 (10.7–4948) ^2^	4.30 ± 0.44	
			Finerenone 10 mg	98 (77/21)	64.94 ± 9.62	67.0 ± 20.9	249.5 (30.4–3917) ^2^	4.30 ± 0.42	
			Finerenone 15 mg	125 (98/27)	63.95 ± 8.34	67.5 ± 23.6	161.1 (21.2–4144) ^2^	4.30 ± 0.46	
			Finerenone 20 mg	119 (89/30)	64.70 ± 9.26	66.0 ± 22.2	202.7 (4.4–2298) ^2^	4.30 ± 0.44	
Pitt B et al. (27)	2021	International	Placebo	3666 (2577/1089)	64.1 ± 10.0	68.0 ± 21.7	315 (111–731) ^1^	4.33 ± 0.43	3.4 years
			Finerenone 10 or 20 mg	3686 (2528/1158)	64.1 ± 9.7	67.6 ± 21.7	302 (105–749) ^1^	4.33 ± 0.43	
Katayama S et al. (28)	2017	Japan	Placebo	12 (10/2)	66.75 ± 9.02	60.88 ± 16.53	287.74 (28.9–1338.1) ^2^	4.21 ± 0.18	90 days
			Finerenone 1.25 mg	12 (9/3)	64.33 ± 9.04	70.23 ± 11.37	191.93 (19.5–605.1) ^2^	4.08 ± 0.27	
			Finerenone 2.5 mg	12 (8/4)	62.67 ± 9.17	60.88 ± 16.36	144.35 (45.1–999.8) ^2^	4.01 ± 0.32	
			Finerenone 5 mg	12 (8/4)	58.08 ± 13.08	67.48 ± 12.16	235.18 (46.0–1893.0) ^2^	4.04 ± 0.29	
			Finerenone 7.5 mg	12 (10/2)	63.17 ± 10.76	62.64 ± 16.89	517.76 (80.5–1203.5) ^2^	4.36 ± 0.50	
			Finerenone 10 mg	12 (12/0)	62.75 ± 7.05	69.79 ± 12.17	260.38 (9.4–2404.4) ^2^	4.42 ± 0.30	
			Finerenone 15 mg	12 (12/0)	61.83 ± 11.17	64.12 ± 14.72	228.67 (51.3–1917.4) ^2^	4.07 ± 0.38	
			Finerenone 20 mg	12 (8/4)	64.00 ± 8.26	61.48 ± 11.01	127.67 (58.6–1356.4) ^2^	4.28 ± 0.36	

eGFR, estimated glomerular filtration rate. ^1^ data was presented as median (interquartile range), ^2^ data was presented as median (range).

**TABLE 2:** Ongoing large trial of finerenone for diabetes patients with CKD.

Registration	Name	Design	Location	Participants	Dose	Duration	Primary outcome
NCT05901831 (30)	FINE-ONE	Double-blind phase III RCT	International	440 type 1 diabetes with CKD	10 or 20 mg	7.5 months	Relative change in UACR from baseline over 6 months
NCT05887817; jRCTs021230011 (31)	FIVE-STAR	Multicenter, prospective, placebo-controlled, double-blind, RCT	Japan	100 type 2 diabetes with CKD	10 mg	24 weeks	Change from baseline in CAVI at 24 weeks
NCT05348733 (32)	FINE-REAL	Prospective, multicenter, single-arm study	International	5500 type 2 diabetes with CKD	10 or 20 mg	12 months	Describe treatment patterns in patients with CKD and type 2 diabetes treated with finerenone in routine clinical practice.
NCT05254002 (33)	CONFIDENCE	Double-blind, double-dummy, international, multicenter, three-armed, parallel-group RCT	International	807 type 2 diabetes with stage 2–3 CKD	10 or 20 mg	6 months	relative change from baseline in UACR

CKD, chronic kidney disease; CAVI, cardio ankle vascular index; UACR, urine albumin/creatinine ratio; RCT, randomized clinical trial.

### Primary outcome: all-cause mortality

**FIGURE 2:**
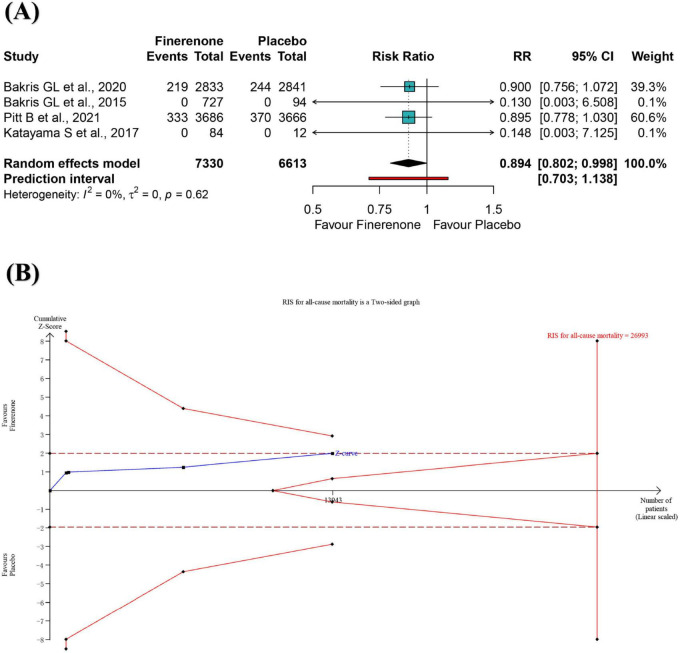
Meta-analysis **(A)** and trial sequential analysis **(B)** of finerenone versus control group for all-cause mortality. RR, ratio risk; TSA, trial sequential analysis; 95% CI, 95% confidence interval.

A meta-analysis of four RCTs showed a significant 10.6% reduction of all-cause mortality risk for participants in the finerenone group compared to placebo group (RR: 0.894; 95% CI 0.802–0.998; I^2^ = 0%) (Figure 2A), with high-certainty evidence (Supplementary Table S4). From this result, the number need to treat required to benefit from finerenone compared to placebo was 56. The 95% prediction interval ranged from 0.703 to 1.138, suggesting that finerenone treatment may not reduce the risk of death in DKD patients compared with placebo in future studies in similar settings (Figure 2A). The informative amount of all-cause mortality in the trial sequence analysis did not meet the required magnitude of 10% relative risk reduction (Figure 2B). A positive effect of similar magnitude was found for “leave-one-out” sensitivity analysis but with no statistical significance (Supplementary Figure S2). The results were not significantly different after excluding two “double zero event” studies (Supplementary Figure S3).

### Secondary outcome: cardiovascular mortality

**FIGURE 3:**
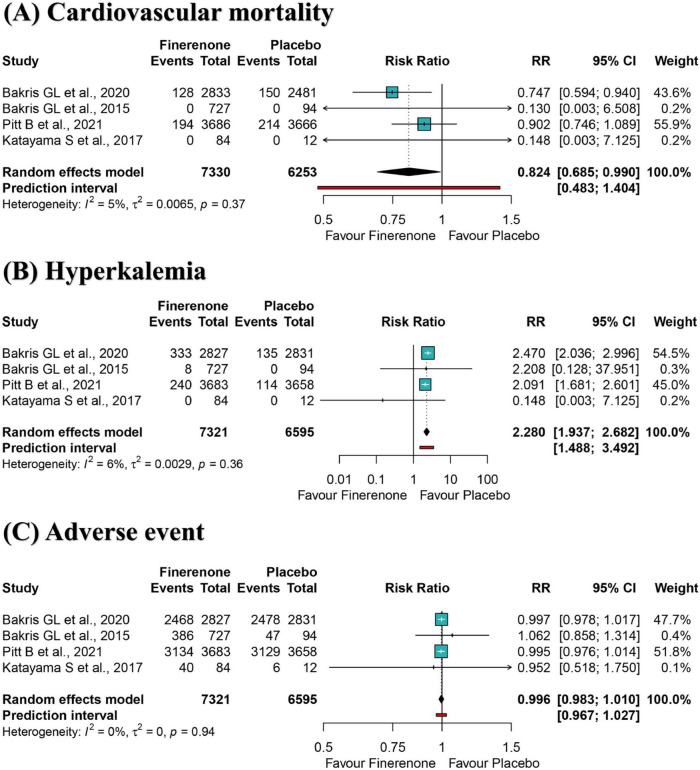
Meta-analysis of finerenone versus control group for cardiovascular mortality **(A)**, hyperkalemia **(B)**, and adverse event **(C)**. RR, ratio risk; 95% CI, 95% confidence interval.

A meta-analysis of four RCTs showed a 17.6% reduction of cardiovascular mortality risk for participants in the finerenone group compared to the placebo group (RR: 0.824; 95% CI 0.685–0.990; I^2^ = 5%) (Figure 3A), with high-certainty evidence (Supplementary Table S4). The 95% prediction intervals ranged from 0.483 to 1.404, suggesting that in future studies in a similar setting, treatment with finerenone, compared to placebo, may not reduce the risk of cardiovascular death in DKD patients (Figure 3A). The informative amount of cardiovascular mortality in the trial sequence analysis did not meet the required magnitude of 10% relative risk reduction (Supplementary Figure S4). Positive effects of similar magnitude were found for “leave-one-out” sensitivity analysis (Supplementary Figure S5). The effect size remained statistically significant after excluding “double-zero event” studies (Supplementary Figure S6).

### Secondary outcome: hyperkalemia

A meta-analysis of four RCTs showed that participants in the finerenone group had a significantly higher risk of developing hyperkalemia than those in the placebo group (RR: 2.280; 95% CI 1.937–2.682; I^2^ = 0%) (Figure 3B), with high-certainty evidence (Supplementary Table S4). The 95% prediction interval ranged from 1.488 to 3.492, suggesting that finerenone treatment is susceptible to hyperkalemia compared to placebo in future studies in a similar setting (Figure 3B). The informative amount of hyperkalemia in the trial sequence analysis did not meet the required magnitude of 10% relative risk reduction (Supplementary Figure S7). Adverse effects of similar magnitude and significance were found for “leave-one-out” sensitivity analysis (Supplementary Figure S8) and excluding “double-zero event” studies (Supplementary Figure S9).

### Secondary outcome: any adverse event

A meta-analysis of four RCTs showed that the risk of any adverse event between the fenugreek and placebo group was not statistically significant (RR: 0.996; 95% CI 0.983–1.010; I^2^ = 0%) (Figure 3C), with high-certainty evidence (Supplementary Table S4). The 95% prediction interval ranged from 0.967 to 1.027, suggesting that both would have a comparable risk of an adverse event in future studies in a similar setting. The informative amount of any adverse event in the trial sequence analysis did not meet the required magnitude of 5% relative risk reduction (Supplementary Figure S10). Effect sizes of similar magnitude and significance were found for “leave-one-out” sensitivity analysis (Supplementary Figure S11).

## Discussion

This review provides an evidence-based summary of four RCTs regarding the prognosis and safety of finerenone versus placebo in DKD patients. The current primary outcome on all-cause mortality is similar to the two previous systematic reviews (11, 12). A 2022 meta-analysis of three RCTs found that finerenone reduced the risk of all-cause mortality relative to controls (RR: 0.90; 95% CI 0.82–0.99) (11). However, the inclusion in this meta-analysis of a RCT of finerenone compared with eplerenone in patients with chronic heart failure, diabetes, and/or CKD (34), was contrary to the purpose of our study. A similar meta-analysis by Yang et al. which included three trials with 13,852 participants and corrected for the continuity of zero events with a “1,” also showed a positive effect of finerenone versus placebo on all-cause mortality risk (12). Compared with these reviews, we added a small sample study from Mineralocorticoid Receptor Antagonist Tolerability Study-diabetic nephropathy (ARTS-DN) Japan (*n* = 96) (28). In addition, our study calculated the number need to treat and showed that finerenone treatment of 56 patients with DKD prevented one all-cause mortality outcome. Nevertheless, the “zero-event” study may have influenced our positive results, and the conclusion that finerenone is associated with a reduced risk of death in DKD patients needs to be further validated in well-designed RCTs.

Mortality is the most important clinical outcome (35). However, the current study sizes do not meet the optimal sample size for a 10% relative risk reduction, and the pooled risk ratio is close to 1 with narrow confidence intervals. Pooled analyses of two large RCTs [FInerenone in reducing kiDnEy faiLure and dIsease prOgression in Diabetic Kidney Disease (FIDELIO-DKD) and FInerenone in reducinG cArdiovascular moRtality and mOrbidity in Diabetic Kidney Disease (FIGARO-DKD)] suggest that finerenone reduces all-cause mortality (16). In the FIDELIO-DKD study, within the first 4 months, subjects experienced a rapid decline in estimated glomerular filtration rate of −3.18 mL/min/1.73 m^2^; however, it began to slow to −2.66 mL/min/1.73 m^2^ thereafter (27). The effect of slowing down renal function decline became progressively more pronounced when finerenone was continued for more than 2 years, and it became more pronounced if it continued for a longer period (10). Evidence shows that slowly declining individuals are strongly associated with a lower risk of all-cause mortality than rapidly declining renal function (36).

In line with the results for all-cause mortality, this study found that finerenone reduced cardiovascular mortality relative to placebo. The results of previous reviews on cardiovascular mortality have been inconsistent. The meta-analysis by Zhu et al. found no evidence that finerenone reduced cardiovascular mortality (RR: 0.88; 95% CI 0.76–1.01) (11). Our findings on cardiovascular mortality are consistent with those of Yang et al. who excluded two “zero-event” studies (12). A similar approach was also found to be significant in our study. In addition, we explored the optimal sample size by trial sequential analysis.

Like several previous meta-analyses (12–14), we also found that finerenone caused elevated serum potassium. A review of randomized studies suggests that mineralocorticoid receptor antagonists further reduce proteinuria in patients with diabetes mellitus or non-diabetes mellitus causes of CKD when combined with renin-angiotensin system blockers (37). However, eplerenone and spironolactone increase the risk of hyperkalemia by 3- 8-fold in patients with stage 3 or more CKD (38). Of note, although the incidence of hyperkalemia was slightly higher in finerenone group than placebo group, not many patients permanently discontinued the drug due to hyperkalemia (122/7331≈1.66%), which is significantly lower than the data on renin-angiotensin system blockers (39). Clinical studies tend to focus on group data, but more attention is paid to individual data in clinical practice. Considering that patients with DKD are at high risk for hyperkalemia, clinicians should be aware and regularly check serum potassium levels when prescribing finerenone.

An ongoing series of large trials designed to evaluate the renoprotective effects of finerenone has the potential to confirm or refute our findings. The results of FINE-ONE (NCT05901831) may expand the kidney-protective indications for finerenone from type 2 diabetes to type 1 diabetes (30), while “FInerenone, in addition to standard of care, on the progression of kidney disease in patients with Non-Diabetic Chronic Kidney Disease” (FIND-CKD, NCT05047263) (40) and FIONA (NCT05196035) (41, 42) may identify a potentially expanded role for finerenone in treating CKD of diabetic and non-diabetic etiology in all age groups. Although FINE-REAL (NCT05348733) is not a RCT, the single-arm trial will enroll approximately 5,500 adults with CKD and type 2 diabetes from 22 countries, and the results will provide insights into the population characteristics and treatment patterns of those treated with finerenone in routine clinical practice (32). The trial is expected to complete enrollment in September 2027. The “Finerenone on Vascular Stiffness and Cardiorenal Biomarkers in Type 2 Diabetes and Chronic Kidney Disease” (FIVE-STAR, NCT05887817) proposes to enroll 100 patients with type 2 diabetes and CKD from 13 centers in Japan aimed to evaluate the effect of finerenone on vascular stiffness (31). Recruitment for this trial has been initiated in September 2023 and is expected to be completed by July 2024. The “COmbinatioN effect of FInerenone anD EmpaglifloziN” (CONFIDENCE, NCT05254002) is a randomized, controlled, double-blind, double-dummy, international, multicenter, three-armed, parallel-group, Phase 2 study, which will illustrate whether dual therapy with finerenone and empagliflozin can provide additional benefits for patients with CKD and type 2 diabetes (33). As it stands, finerenone has been studied primarily in patients with type 2 diabetes combined with CKD, so the currently approved indication is also for this population. As those studies progress, finerenone is expected to expand its use in other types of kidney disease further (43).

This systematic review and meta-analysis have several methodological strengths. We followed the recommendations of the Cochrane Collaboration and the PRISMA statement, including *a priori* protocols. This study also used the GRADE method to assess the certainty of evidence and the minimum amount of information required by trial sequence analysis. Our study has some important limitations. First, no deaths were reported in two studies of short duration, potentially distorting the effect size of the primary outcome. Pooled analyses that included only two studies corroborated our concerns. Second, all-cause mortality reported in all included trials was a secondary outcome of the trials. The way in which secondary outcome data are collected may differ from how primary outcome data are collected. Third, this meta-analysis was not tested for publication bias due to the limitations of the included studies, but two included studies may have had a slight sample bias that exaggerated the existing findings. Fourth, further research for dose-response analysis and compatibility of finerenone is needed. Fifth, only four studies were included in this meta-analysis, meaning that relatively little pooled data are available, so results must be interpreted cautiously. Fortunately, there are a growing number of ongoing trials, and the results of these studies may contribute to our understanding of the therapeutic effects of finerenone in DKD patients.

## Conclusion

Overall, finerenone was associated with an 10.6% reduction in all-cause mortality and a 17.6% reduction in cardiovascular mortality in DKD patients relative to placebo, respectively. However, finerenone is prone to hyperkalemia. Further studies are needed to validate the prognosis of finerenone among patients with DKD.

## Data Availability

The original contributions presented in this study are included in this article/Supplementary material, further inquiries can be directed to the corresponding authors.

## Footnotes

1
https://classic.clinicaltrials.gov/

2
apps.who.int/trialsearch/Default.aspx

3
https://www.riskofbias.info/welcome
