# Supplementary material for: Association of finerenone with prognosis and safety in diabetic kidney disease patients: an undated meta-analysis based on four RCTs
Source: Front Med (Lausanne). 2025 Jul 3;12:1594202. doi: 10.3389/fmed.2025.1594202 (PMC12267240; doi:10.3389/fmed.2025.1594202)
Supplement: Supplementary file 1 [file Data_Sheet_1.docx]

**Association of finerenone with prognosis and safety in diabetic kidney disease patients: an undated meta-analysis based on four RCTs.**

**Supplementary Material**

**Content**

[Table S1 The 2020 PRISMA Checklist. 2](#_Toc157950542)

[Table S2 Protocol deviations. 6](#_Toc157950543)

[Table S3 Search detailed for databases. 7](#_Toc157950544)

[Table S4 GRADE evidence profile. 10](#_Toc157950545)

[Figure S1 Risk of bias for including studies. 12](#_Toc157950546)

[Figure S2 Sensitivity analysis of all-cause mortality risk based on "leave one out" approach. 13](#_Toc157950547)

[Figure S3 Forest plot of all-cause mortality risk excluding "double-zero events". 14](#_Toc157950548)

[Figure S4 Trial sequential analysis of finerenone versus control group for cardiovascular mortality. 15](#_Toc157950549)

[Figure S5 Sensitivity analysis of cardiovascular mortality risk based on "leave one out" approach. 16](#_Toc157950550)

[Figure S6 Forest plot of cardiovascular mortality risk excluding "double-zero events". 17](#_Toc157950551)

[Figure S7 Trial sequential analysis of finerenone versus control group for hyperkalemia. 18](#_Toc157950552)

[Figure S8 Sensitivity analysis of hyperkalemia risk based on "leave one out" approach. 19](#_Toc157950553)

[Figure S9 Forest plot of hyperkalemia risk excluding "double-zero events". 20](#_Toc157950554)

[Figure S10 Trial sequential analysis of finerenone versus control group for adverse event. 21](#_Toc157950555)

[Figure S11 Sensitivity analysis of adverse event risk based on "leave one out" approach. 22](#_Toc157950556)

Table S1 The 2020 PRISMA Checklist.

| **Section and Topic** | **Item #** | **Checklist item** | **Location where item is reported** |
| --- | --- | --- | --- |
| **TITLE** | | |  |
| Title | 1 | Identify the report as a systematic review. | Title page |
| **ABSTRACT** | | |  |
| Abstract | 2 | See the PRISMA 2020 for Abstracts checklist. | Abstract |
| **INTRODUCTION** | | |  |
| Rationale | 3 | Describe the rationale for the review in the context of existing knowledge. | Page 4 |
| Objectives | 4 | Provide an explicit statement of the objective(s) or question(s) the review addresses. | Page 4-5 |
| **METHODS** | | |  |
| Eligibility criteria | 5 | Specify the inclusion and exclusion criteria for the review and how studies were grouped for the syntheses. | Page 5-6 |
| Information sources | 6 | Specify all databases, registers, websites, organisations, reference lists and other sources searched or consulted to identify studies. Specify the date when each source was last searched or consulted. | Page 5 |
| Search strategy | 7 | Present the full search strategies for all databases, registers and websites, including any filters and limits used. | Table S3 |
| Selection process | 8 | Specify the methods used to decide whether a study met the inclusion criteria of the review, including how many reviewers screened each record and each report retrieved, whether they worked independently, and if applicable, details of automation tools used in the process. | Page 6 |
| Data collection process | 9 | Specify the methods used to collect data from reports, including how many reviewers collected data from each report, whether they worked independently, any processes for obtaining or confirming data from study investigators, and if applicable, details of automation tools used in the process. | Page 6 |
| Data items | 10a | List and define all outcomes for which data were sought. Specify whether all results that were compatible with each outcome domain in each study were sought (e.g. for all measures, time points, analyses), and if not, the methods used to decide which results to collect. | Page 6 |
|  | 10b | List and define all other variables for which data were sought (e.g. participant and intervention characteristics, funding sources). Describe any assumptions made about any missing or unclear information. | Table 1 |
| Study risk of bias assessment | 11 | Specify the methods used to assess risk of bias in the included studies, including details of the tool(s) used, how many reviewers assessed each study and whether they worked independently, and if applicable, details of automation tools used in the process. | Page 7 |
| Effect measures | 12 | Specify for each outcome the effect measure(s) (e.g. risk ratio, mean difference) used in the synthesis or presentation of results. | Page 7 |
| Synthesis methods | 13a | Describe the processes used to decide which studies were eligible for each synthesis (e.g. tabulating the study intervention characteristics and comparing against the planned groups for each synthesis (item #5)). | Page 6-7 |
|  | 13b | Describe any methods required to prepare the data for presentation or synthesis, such as handling of missing summary statistics, or data conversions. | Page 6-7 |
|  | 13c | Describe any methods used to tabulate or visually display results of individual studies and syntheses. | Page 6-7 |
|  | 13d | Describe any methods used to synthesize results and provide a rationale for the choice(s). If meta-analysis was performed, describe the model(s), method(s) to identify the presence and extent of statistical heterogeneity, and software package(s) used. | Page 6-7 |
|  | 13e | Describe any methods used to explore possible causes of heterogeneity among study results (e.g. subgroup analysis, meta-regression). | Page 6-7 |
|  | 13f | Describe any sensitivity analyses conducted to assess robustness of the synthesized results. | Page 5-6 |
| Reporting bias assessment | 14 | Describe any methods used to assess risk of bias due to missing results in a synthesis (arising from reporting biases). | Page 6-7 |
| Certainty assessment | 15 | Describe any methods used to assess certainty (or confidence) in the body of evidence for an outcome. | Page 7 |
| **RESULTS** | | |  |
| Study selection | 16a | Describe the results of the search and selection process, from the number of records identified in the search to the number of studies included in the review, ideally using a flow diagram. | Page 7 and Figure 1 |
|  | 16b | Cite studies that might appear to meet the inclusion criteria, but which were excluded, and explain why they were excluded. | Figure 1 |
| Study characteristics | 17 | Cite each included study and present its characteristics. | Table 1 |
| Risk of bias in studies | 18 | Present assessments of risk of bias for each included study. | Figure S1 |
| Results of individual studies | 19 | For all outcomes, present, for each study: (a) summary statistics for each group (where appropriate) and (b) an effect estimate and its precision (e.g. confidence/credible interval), ideally using structured tables or plots. | Figure 2 and Figure 3 |
| Results of syntheses | 20a | For each synthesis, briefly summarise the characteristics and risk of bias among contributing studies. | Page 13-14 |
|  | 20b | Present results of all statistical syntheses conducted. If meta-analysis was done, present for each the summary estimate and its precision (e.g. confidence/credible interval) and measures of statistical heterogeneity. If comparing groups, describe the direction of the effect. | Page 13-14 |
|  | 20c | Present results of all investigations of possible causes of heterogeneity among study results. | Not applicable |
|  | 20d | Present results of all sensitivity analyses conducted to assess the robustness of the synthesized results. | Page 13-14 |
| Reporting biases | 21 | Present assessments of risk of bias due to missing results (arising from reporting biases) for each synthesis assessed. | Page 13-14 |
| Certainty of evidence | 22 | Present assessments of certainty (or confidence) in the body of evidence for each outcome assessed. | Table S4 |
| **DISCUSSION** | | |  |
| Discussion | 23a | Provide a general interpretation of the results in the context of other evidence. | Page 14-15 |
|  | 23b | Discuss any limitations of the evidence included in the review. | Page 17 |
|  | 23c | Discuss any limitations of the review processes used. | Page 17 |
|  | 23d | Discuss implications of the results for practice, policy, and future research. | Page 16-17 |
| **OTHER INFORMATION** | | |  |
| Registration and protocol | 24a | Provide registration information for the review, including register name and registration number, or state that the review was not registered. | Page 5 |
|  | 24b | Indicate where the review protocol can be accessed, or state that a protocol was not prepared. | Page 5 |
|  | 24c | Describe and explain any amendments to information provided at registration or in the protocol. | Table S2 |
| Support | 25 | Describe sources of financial or non-financial support for the review, and the role of the funders or sponsors in the review. | Page 18 |
| Competing interests | 26 | Declare any competing interests of review authors. | Page 18 |
| Availability of data, code and other materials | 27 | Report which of the following are publicly available and where they can be found: template data collection forms; data extracted from included studies; data used for all analyses; analytic code; any other materials used in the review. | Page 18 |

**.**

Table S2 Protocol deviations.

| **Section** | **Previous protocol** | **Publication** | **Reasons** |
| --- | --- | --- | --- |
| Title | Therapeutic effects of finerenone for patients with diabetic kidney disease: a systematic reviews and meta-analyses with trial sequential analysis. | Association of finerenone with prognosis and safety in diabetic kidney disease patients: a meta-analysis based on four randomized placebo-controlled trials. | Our systematic review and meta-analysis preferred to focus on the outcome of finerenone for prognosis and risk of death in patients with diabetic kidney disease |
| Author | Fan Zhang, Zixuan Zhang, Yan Bai, Hui Wang, Liuyan Huang, Yifei Zhong. | Fan Zhang, Zixuan Zhang, Yan Bai, Hui Wang, Liuyan Huang, Yifei Zhong, Yi Li. | Yi Li gave revisions during manuscript review |
| Main outcome | Cardiovascular event. | All-cause mortality. | After discussion during the full-text review process, we focused more on impact of finerenone on mortality outcomes in diabetic nephropathy patients. |
| Additional outcomes | Kidney function and adverse event. | Cardiovascular mortality，hyperkalemia, and adverse event. | Some studies provided renal function continuous results, while others provided categorical results, making meta-analysis impossible. |

Table S3 Search detailed for databases.

| **NO.** | **Search detail** | **Results** |
| --- | --- | --- |
| #1 | "Diabetic Nephropathies"[Mesh] OR "Diabetic Nephropathy"[title/abstract] OR "Diabetic Nephropathies"[title/abstract] OR "Diabetic Kidney Disease"[title/abstract] OR "Diabetic Kidney Diseases"[title/abstract] OR "Diabetic Glomerulosclerosis"[title/abstract] OR "Intracapillary Glomerulosclerosis"[title/abstract] OR "Nodular Glomerulosclerosis"[title/abstract] OR "Kimmelstiel-Wilson Syndrome"[title/abstract] OR "Kimmelstiel Wilson Syndrome"[title/abstract] OR "Kimmelstiel-Wilson Disease"[title/abstract] OR "Kimmelstiel Wilson Disease"[title/abstract] | 40817 |
| #2 | "Finerenone"[title/abstract] OR "BAY 94-8862"[title/abstract] | 399 |
| #3 | #1 AND #2 | 165 |
| 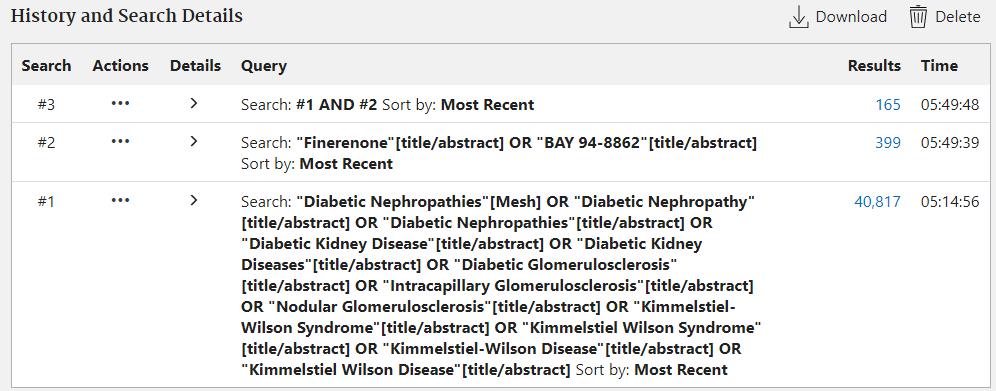 | | |
| **NO.** | **Web of Science** |  |
| #1 | TS=("Diabetic Nephropathy" OR "Diabetic Nephropathies" OR "Diabetic Kidney Disease" OR "Diabetic Kidney Diseases" OR "Diabetic Glomerulosclerosis" OR "Intracapillary Glomerulosclerosis" OR "Nodular Glomerulosclerosis" OR "Kimmelstiel-Wilson Syndrome" OR "Kimmelstiel Wilson Syndrome" OR "Kimmelstiel-Wilson Disease" OR "Kimmelstiel Wilson Disease") | 33456 |
| #2 | TS=("Finerenone" OR "BAY 94-8862") | 553 |
| #3 | #1 AND #2 | 160 |
| 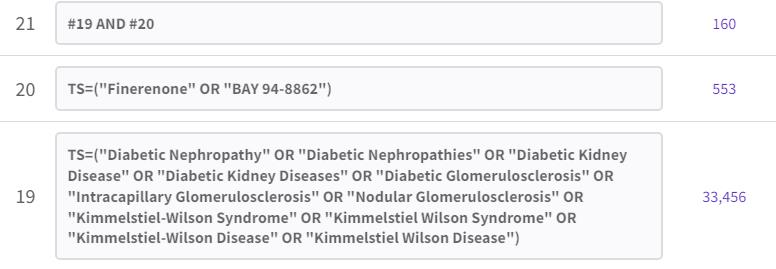 | |  |
| **NO** | **Embase** |  |
| #1 | 'diabetic nephropathy'/exp OR 'Diabetic Nephropathies':ti,ab,kw OR 'Diabetic Kidney Disease':ti,ab,kw OR 'Diabetic Kidney Diseases':ti,ab,kw OR 'Diabetic Glomerulosclerosis':ti,ab,kw OR 'Intracapillary Glomerulosclerosis':ti,ab,kw OR "Nodular Glomerulosclerosis':ti,ab,kw OR 'Kimmelstiel-Wilson Syndrome':ti,ab,kw OR 'Kimmelstiel Wilson Syndrome':ti,ab,kw OR 'Kimmelstiel-Wilson Disease':ti,ab,kw OR 'Kimmelstiel Wilson Disease':ti,ab,kw | 58989 |
| #2 | 'finerenone'/exp OR "Finerenone':ti,ab,kw OR "BAY 94-8862':ti,ab,kw | 951 |
| #3 | #1 AND #2 | 311 |
| 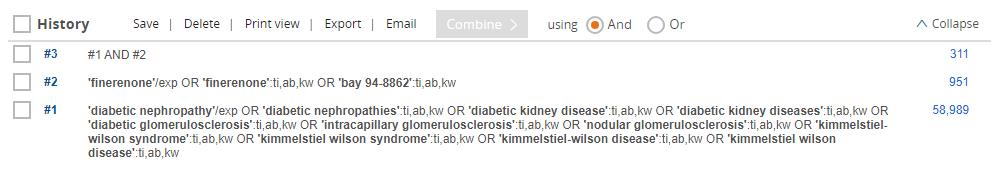 | | |
| NO | Scopus |  |
| #1 | TITLE-ABS-KEY ("Diabetic Nephropathy" OR "Diabetic Nephropathies" OR "Diabetic Kidney Disease" OR "Diabetic Kidney Diseases" OR "Diabetic Glomerulosclerosis" OR "Intracapillary Glomerulosclerosis" OR "Nodular Glomerulosclerosis" OR "Kimmelstiel-Wilson Syndrome" OR "Kimmelstiel Wilson Syndrome" OR "Kimmelstiel-Wilson Disease" OR "Kimmelstiel Wilson Disease") | 60489 |
| #2 | TITLE-ABS-KEY ("Finerenone" OR "BAY 94-8862") | 780 |
| #3 | #1 AND #2 | 321 |
| 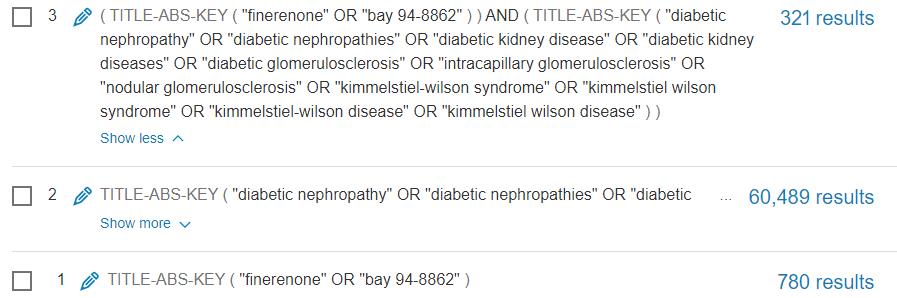 | | |

Table S4 GRADE evidence profile.

| **Certainty assessment** | | | | | | | **№ of patients** | **Effect** | **Certainty** | **Importance** |
| --- | --- | --- | --- | --- | --- | --- | --- | --- | --- | --- |
| **№ of studies** | **Study design** | **Risk of bias** | **Inconsistency** | **Indirectness** | **Imprecision** | **Other considerations** | **[Finerenone vs. placebo]** | **Relative (95% CI)** |  |  |
| **All-cause mortality** | | | | | | | | | | |
| 4 | RCT | Not serious ^a^ | Not serious ^b^ | Not serious ^c^ | Not serious ^d^ | Larger sample size | 7330 vs. 6613 | RR: 0.894; 95% CI 0.802-0.998 | ⨁⨁⨁⨁  High | Important |
| **Cardiovascular mortality** | | | | | | | | | | |
| 4 | RCT | Not serious ^a^ | Not serious ^b^ | Not serious ^c^ | Not serious ^d^ | Larger sample size | 7330 vs. 6253 | RR: 0.824; 95% CI 0.685-0.990 | ⨁⨁⨁⨁  High | Important |
| **Hyperkalemia** | | | | | | | | | | |
| 4 | RCT | Not serious ^a^ | Not serious ^b^ | Not serious ^c^ | Not serious ^e^ | Larger sample size | 7321 vs. 6595 | RR: 2.280; 95% CI 1.937-2.682 | ⨁⨁⨁⨁  High | Important |
| **Adverse event** | | | | | | | | | | |
| 4 | RCT | Not serious ^a^ | Not serious ^b^ | Not serious ^c^ | Not serious ^f^ | Larger sample size | 7321 vs. 6595 | RR: 0.996; 95% CI 0.983-1.010 | ⨁⨁⨁⨁  High | Important |

**Explanation**

a All studies were rated as lower risk.

b *I*^2^<25%.

c All studies reported the effect of finererone versus placebo in diabetes patients with CKD.

d The pooled risk ratio is close to 1 with narrow confidence intervals.

e The effect size did not cross the null line, and the narrower confidence intervals.

f The effect size cross the null line, with a narrower confidence intervals.

Figure S1 Risk of bias for including studies.

**
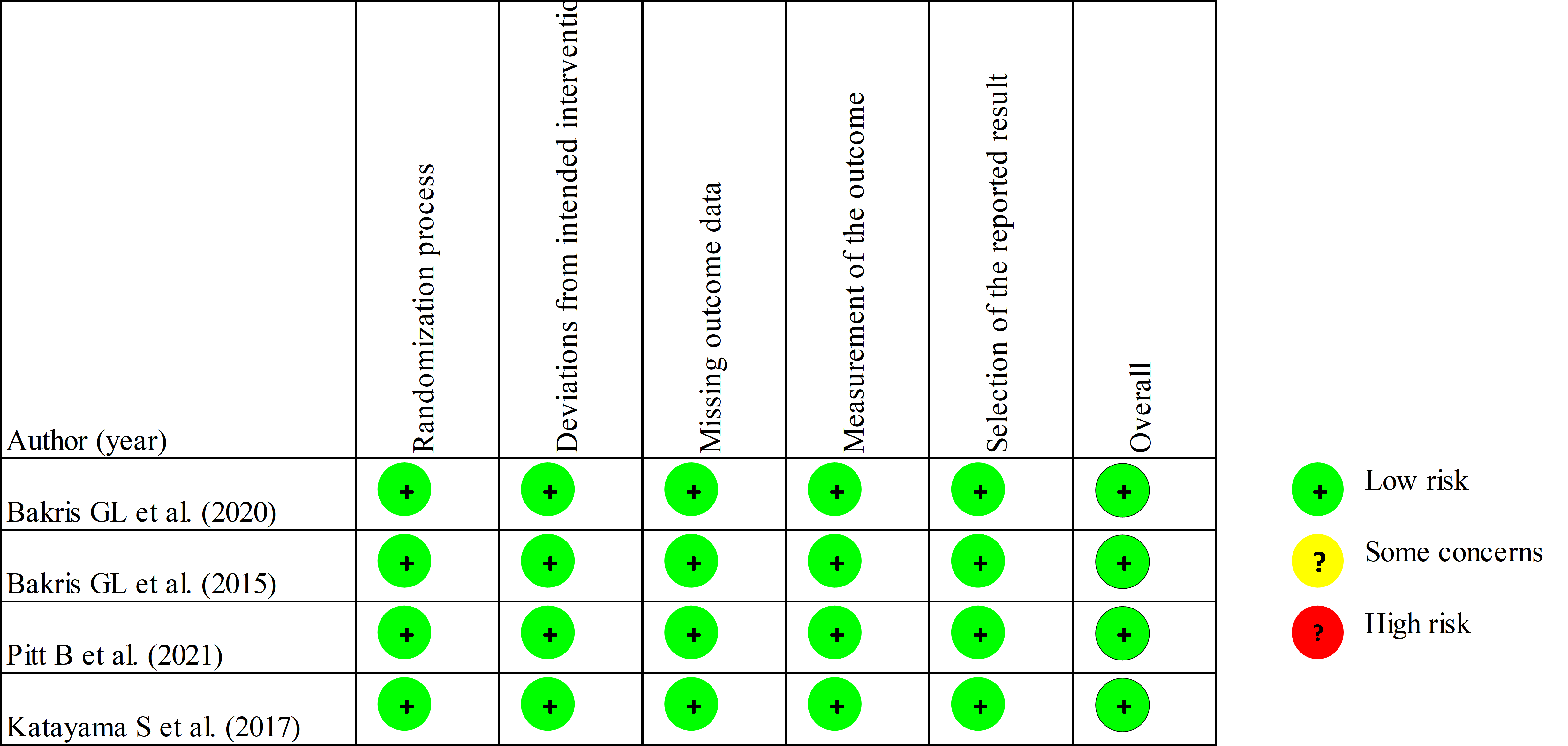
**

Figure S2 Sensitivity analysis of all-cause mortality risk based on "leave one out" approach.

**
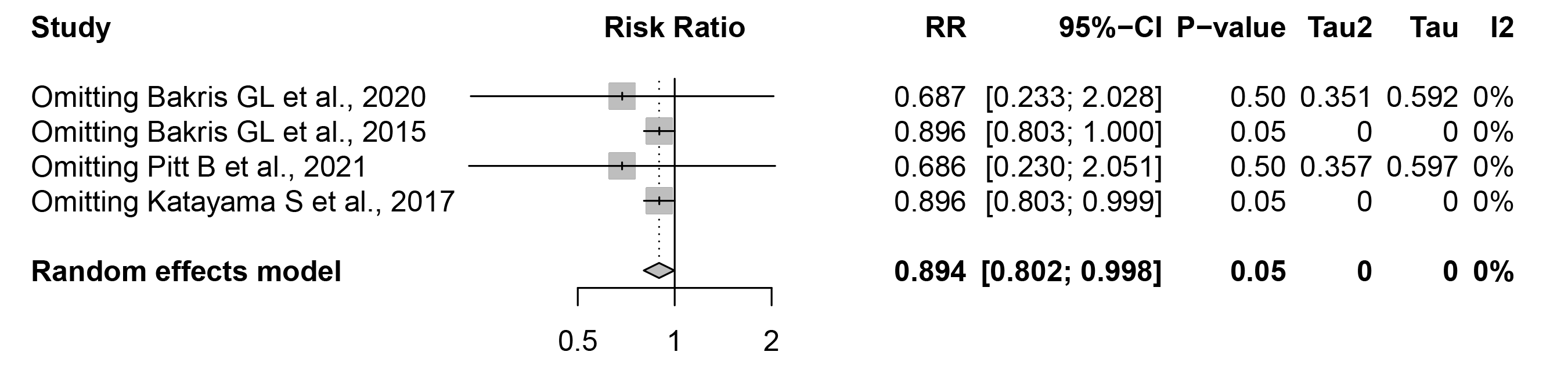
**

Figure S3 Forest plot of all-cause mortality risk excluding "double-zero events".

**
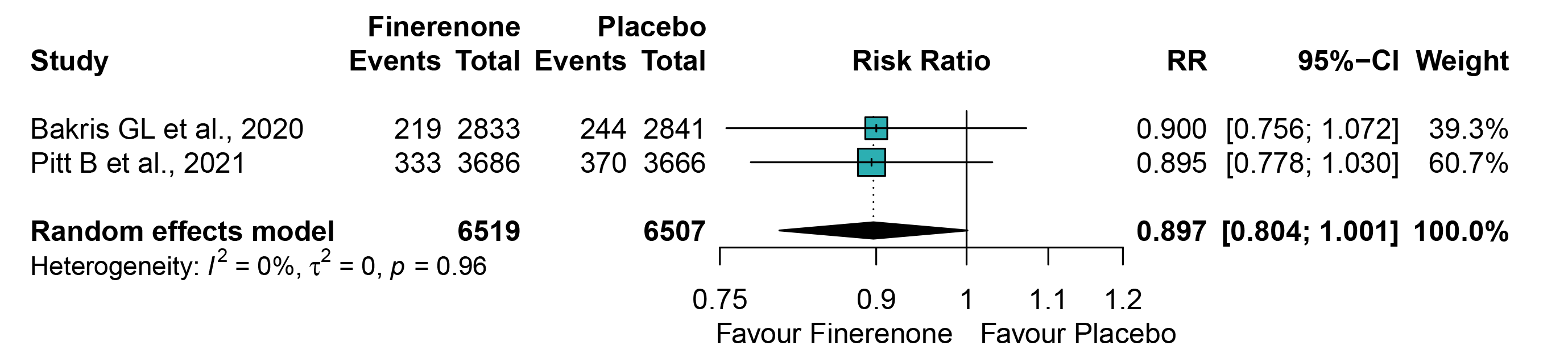
**

Figure S4 Trial sequential analysis of finerenone versus control group for cardiovascular mortality.

**
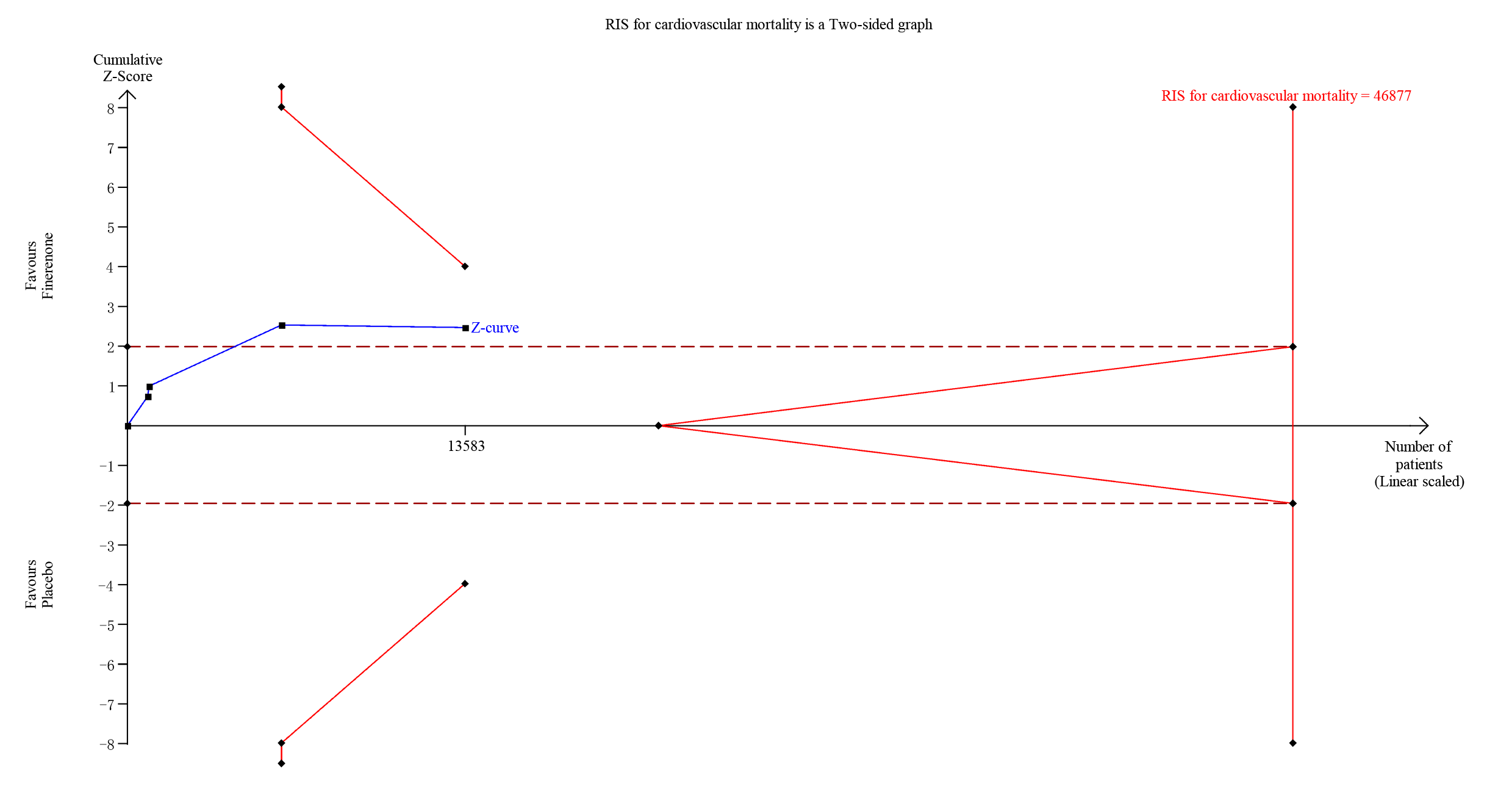
**

Figure S5 Sensitivity analysis of cardiovascular mortality risk based on "leave one out" approach.

**
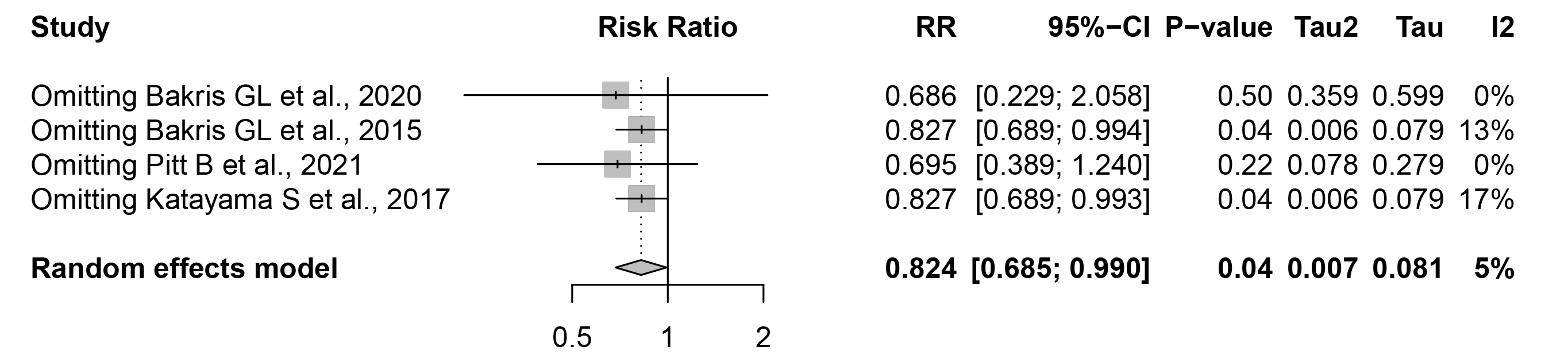
**

Figure S6 Forest plot of cardiovascular mortality risk excluding "double-zero events".

**
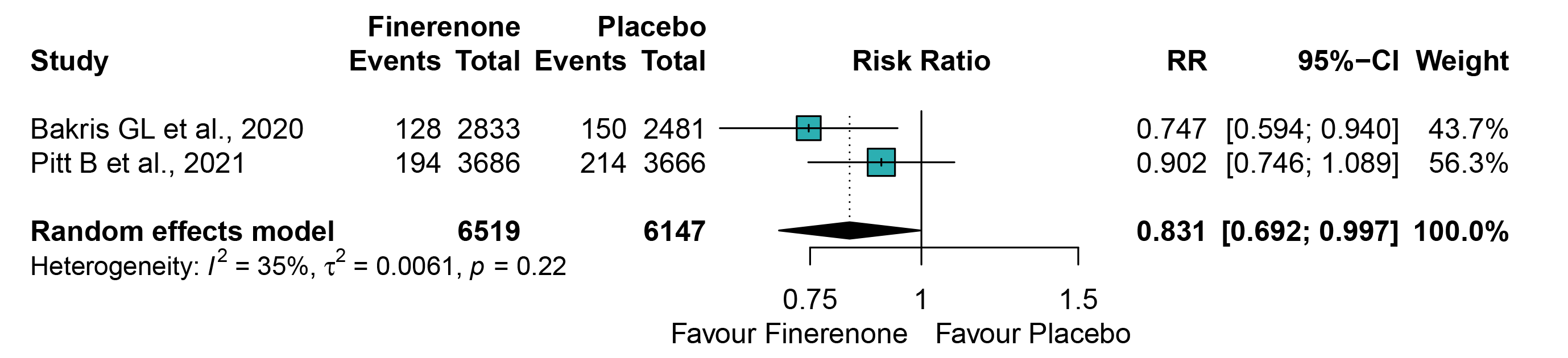
**

Figure S7 Trial sequential analysis of finerenone versus control group for hyperkalemia.

**
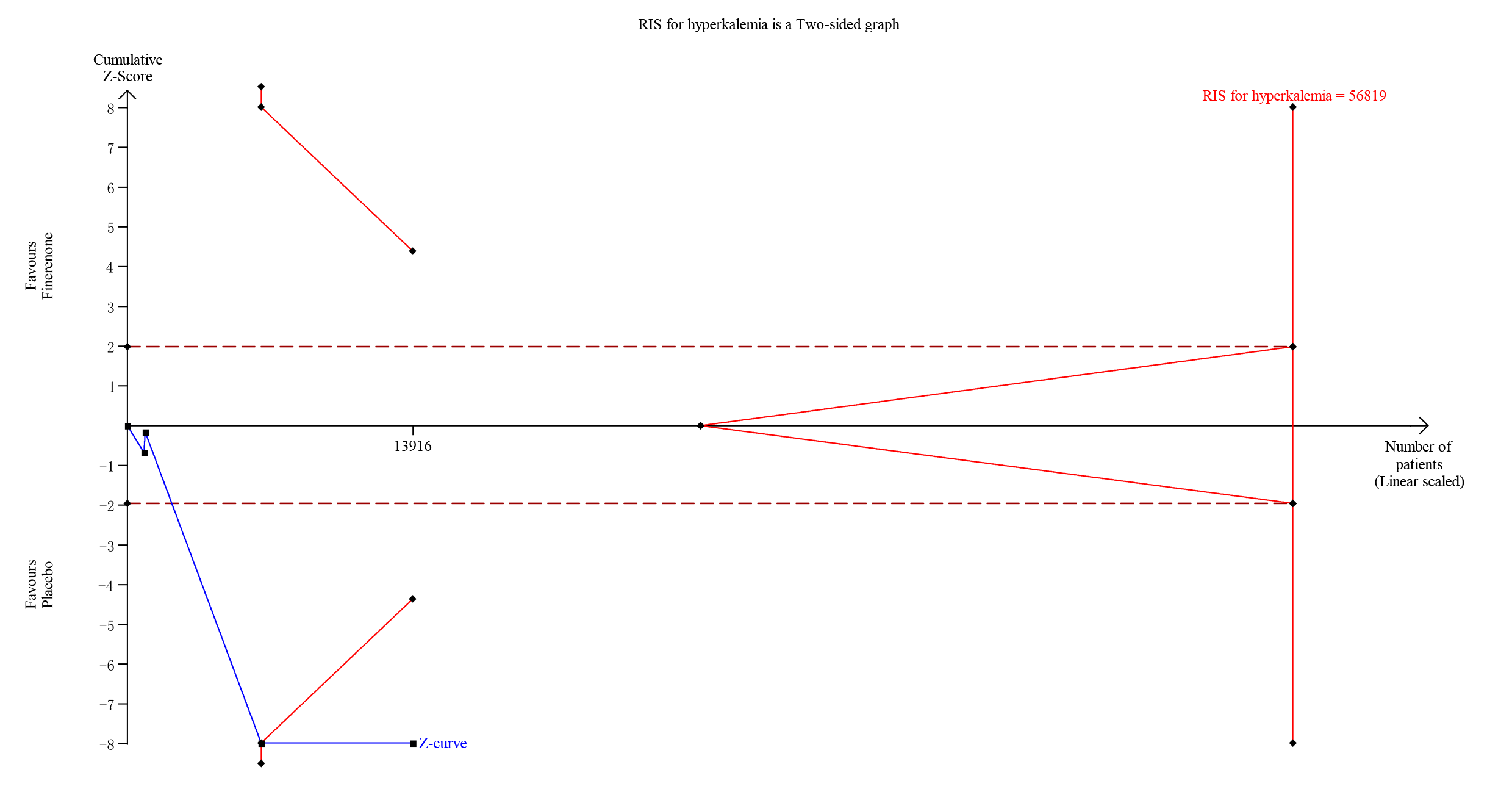
**

Figure S8 Sensitivity analysis of hyperkalemia risk based on "leave one out" approach.

**
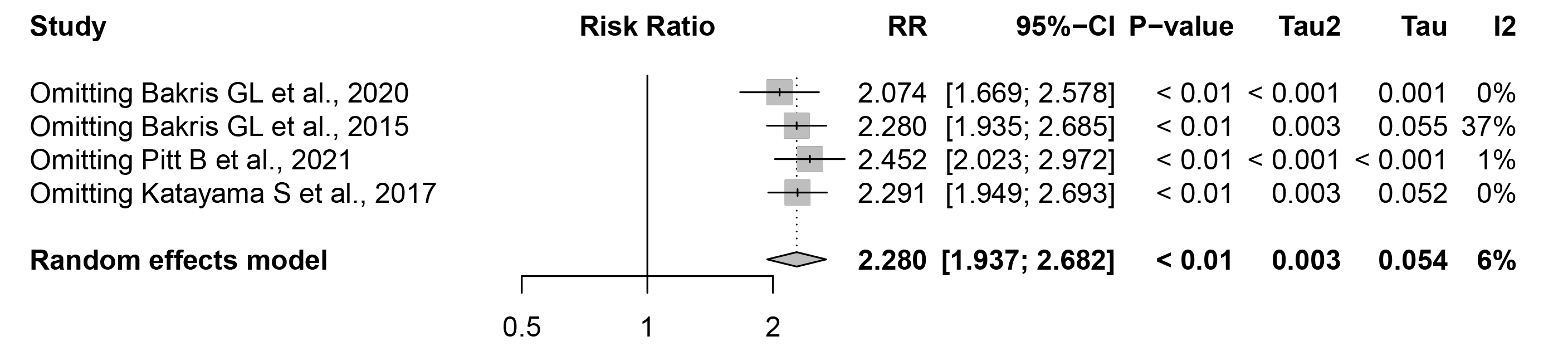
**

Figure S9 Forest plot of hyperkalemia risk excluding "double-zero events".

**
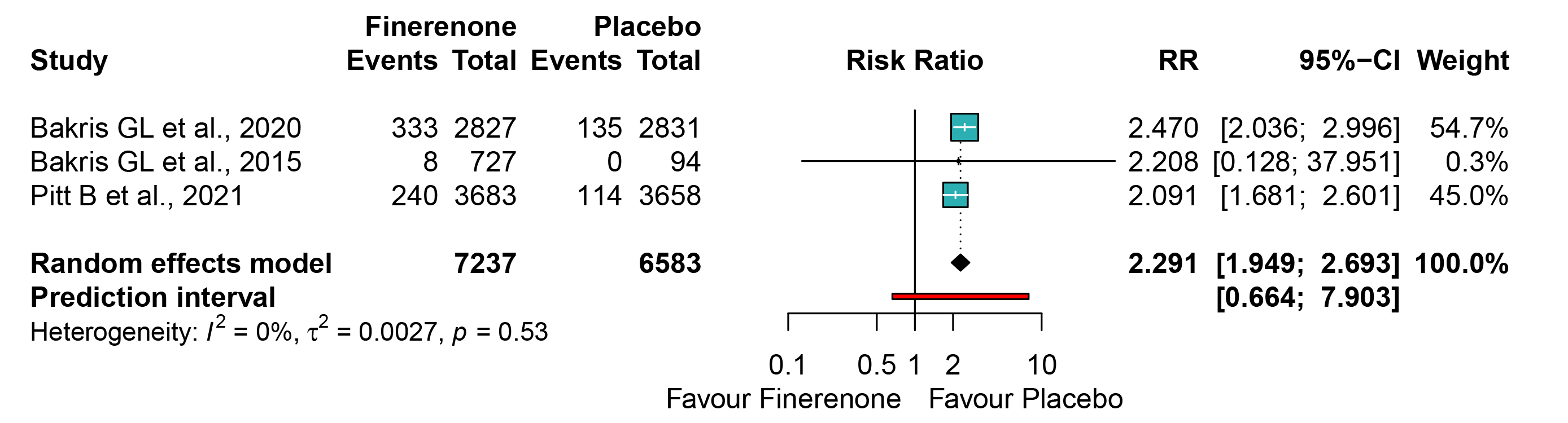
**

Figure S10 Trial sequential analysis of finerenone versus control group for adverse event.

**
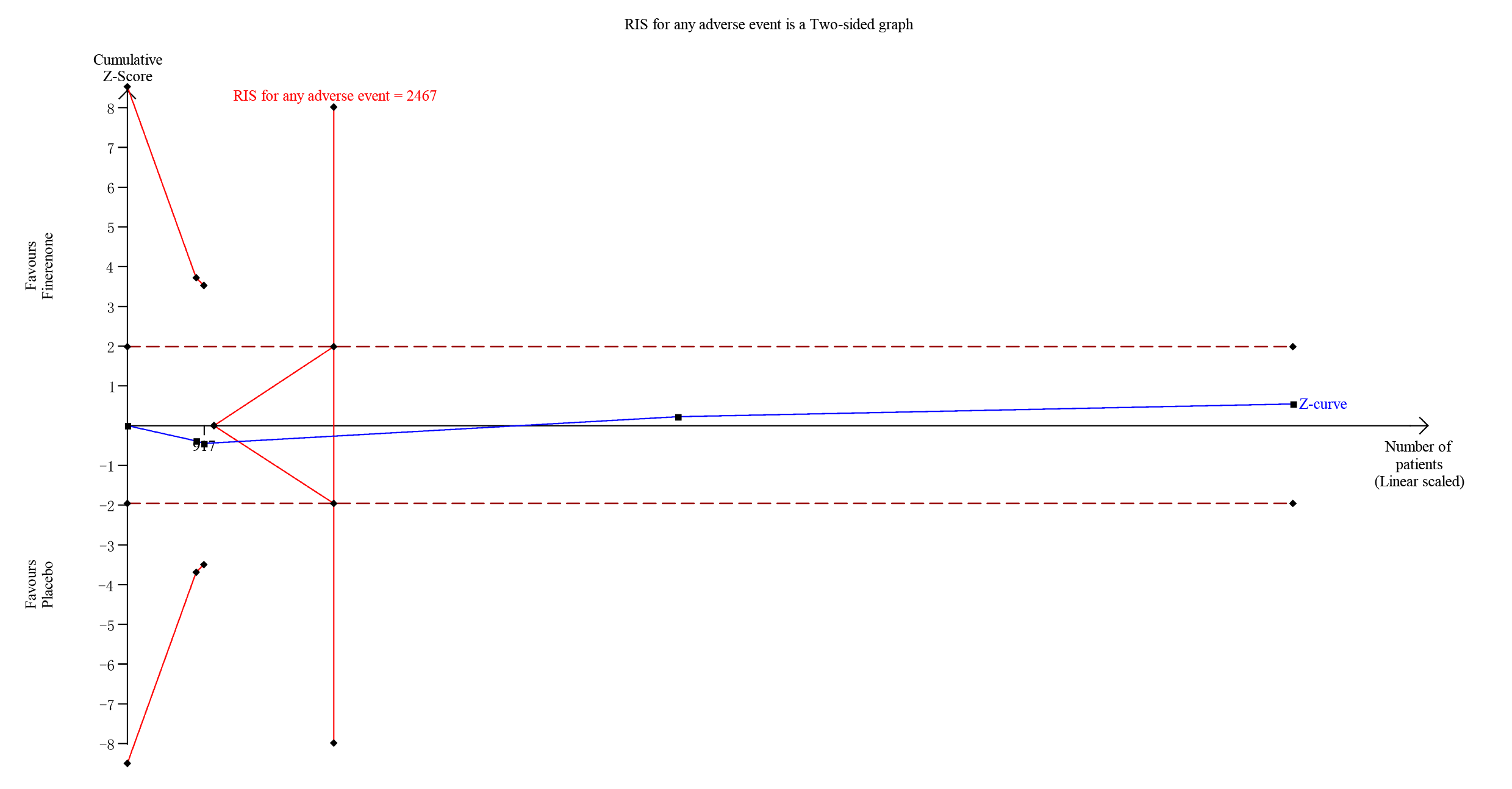
**

Figure S11 Sensitivity analysis of adverse event risk based on "leave one out" approach.

**
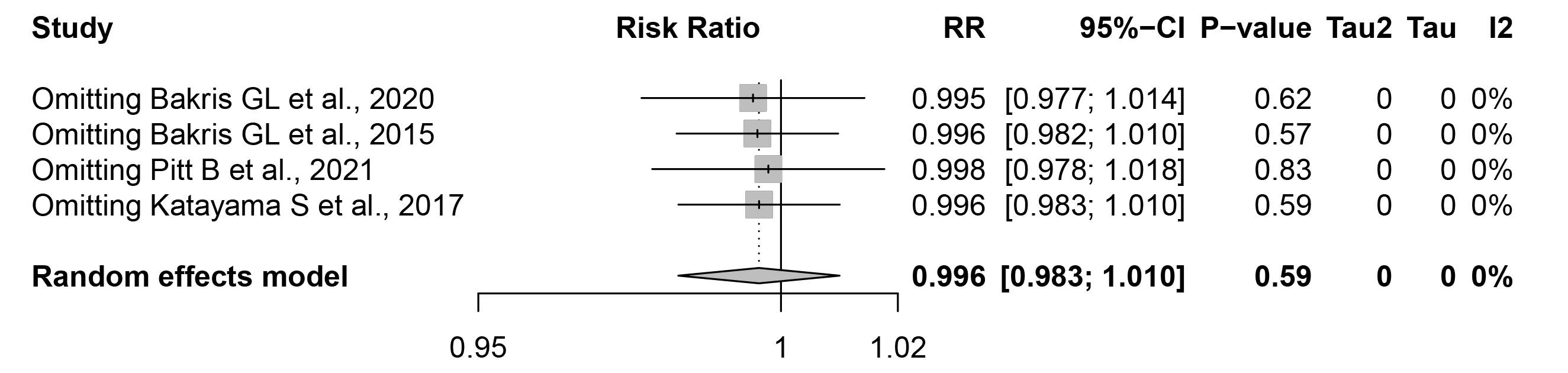
**
